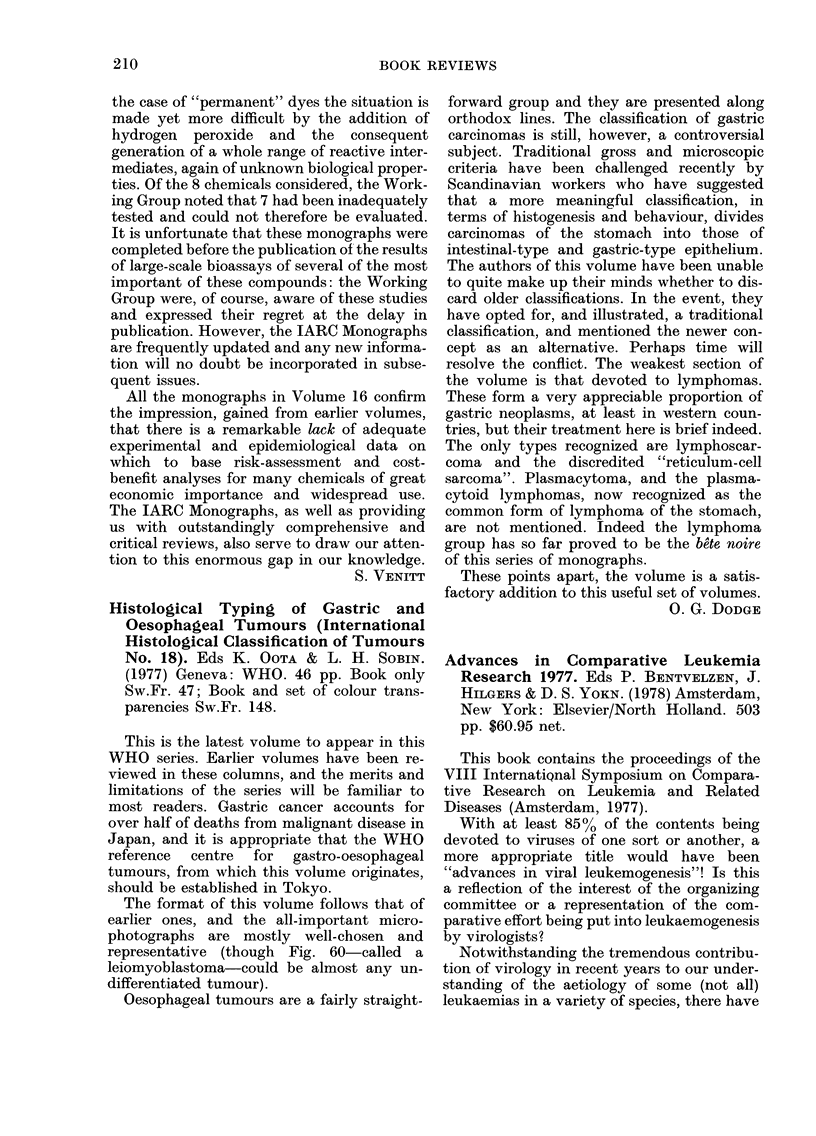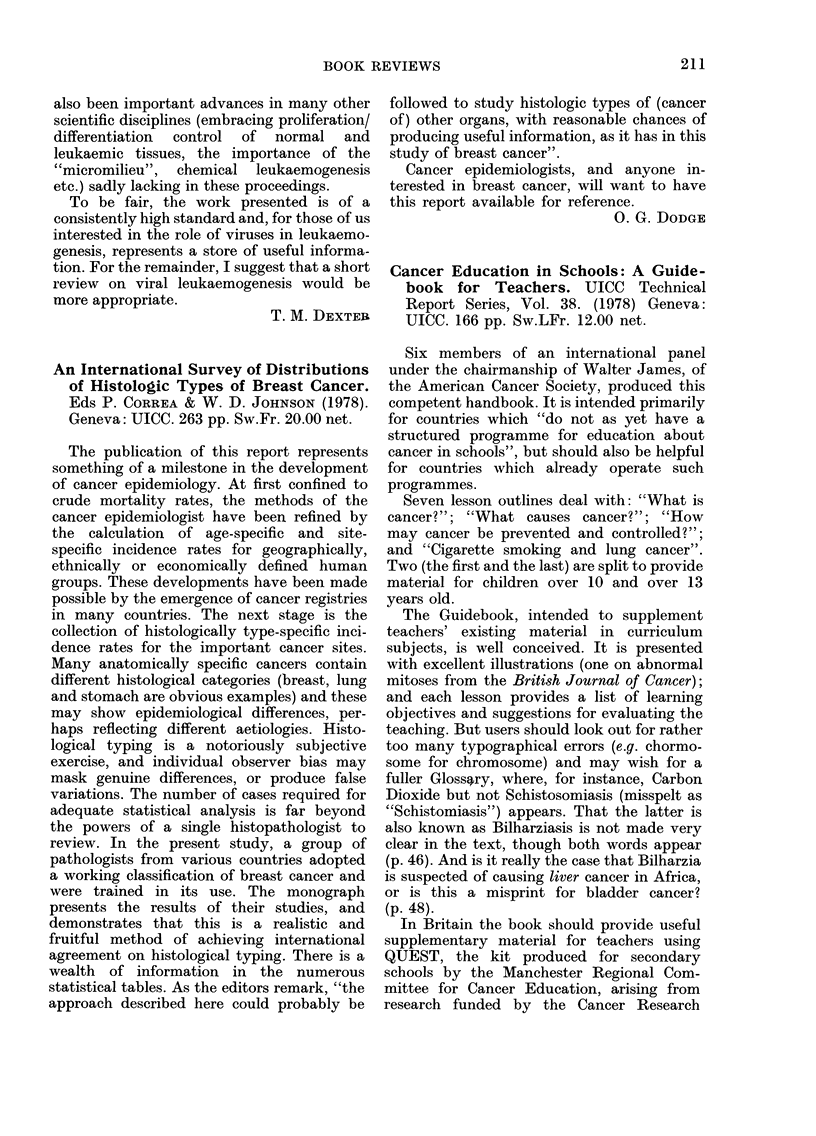# Advances in Comparative Leukemia Research 1977

**Published:** 1979-02

**Authors:** T. M. Dexter


**Advances in Comparative Leukemia Research 1977.** Eds P. Bentvelzen, J. Hilgers & D. S. Yokn. (1978) Amsterdam, New York: Elsevier/North Holland. 503 pp. $60.95 net.

This book contains the proceedings of the VIII International Symposium on Comparative Research on Leukemia and Related Diseases (Amsterdam, 1977).

With at least 85% of the contents being devoted to viruses of one sort or another, a more appropriate title would have been "advances in viral leukemogenesis"! Is this a reflection of the interest of the organizing committee or a representation of the comparative effort being put into leukaemogenesis by virologists?

Notwithstanding the tremendous contribution of virology in recent years to our understanding of the aetiology of some (not all) leukaemias in a variety of species, there have also been important advances in many other scientific disciplines (embracing proliferation/differentiation control of normal and leukaemic tissues, the importance of the "micromilieu", chemical leukaemogenesis etc.) sadly lacking in these proceedings.

To be fair, the work presented is of a consistently high standard and, for those of us interested in the role of viruses in leukaemogenesis, represents a store of useful information. For the remainder, I suggest that a short review on viral leukaemogenesis would be more appropriate.

T. M. DEXTER